# User Beware: Inaccuracy and Inconsistency of Large Language Models in Providing Precision Dosing Recommendations for Patients With Kidney Impairment—A Case Series

**DOI:** 10.1002/phar.70207

**Published:** 2026-10-01

**Authors:** H. Rhodes Hambrick, Samuel Dubinsky, Danica Quickfall, Shannon Reinert, Sonya Tang Girdwood, Gideon Stitt

**Affiliations:** ^1^ Division of Nephrology Ann & Robert H. Lurie Children's Hospital of Chicago Chicago Illinois USA; ^2^ Department of Pediatrics Northwestern University Feinberg School of Medicine Chicago Illinois USA; ^3^ Department of Critical Care Dalhousie University Halifax Nova Scotia Canada; ^4^ Department of Pharmacology Dalhousie University Halifax Nova Scotia Canada; ^5^ Department of Critical Care Mayo Clinic Scottsdale Arizona USA; ^6^ Division of Nephrology and Hypertension Cincinnati Children's Hospital Medical Center Cincinnati Ohio USA; ^7^ Division of Translational and Clinical Pharmacology Cincinnati Children's Hospital Medical Center Cincinnati Ohio USA; ^8^ Division of Hospital Medicine Cincinnati Children's Hospital Medical Center Cincinnati Ohio USA; ^9^ Center for Acute Care Nephrology Cincinnati Children's Hospital Medical Center Cincinnati Ohio USA; ^10^ Department of Pediatrics University of Cincinnati College of Medicine Cincinnati Ohio USA; ^11^ Division of Clinical Pharmacology University of Utah Salt Lake City Utah USA

**Keywords:** acute kidney injury, artificial intelligence, beta lactams, continuous kidney replacement therapy, large language models, pharmacokinetics

## Abstract

**Introduction:**

Evidence‐based dosing guidance for medications in critically ill patients with acute kidney injury (AKI) and receiving continuous kidney replacement therapy (CKRT) is limited. Freely available large language models (LLMs) can generate confident, human‐like outputs. The accuracy and reproducibility of LLMs in providing precision drug dosing recommendations in the setting of AKI and CKRT have not been evaluated.

**Objectives:**

We sought to characterize literature concordance and internal consistency of LLM‐generated dosing recommendations for cefepime and meropenem in patients with AKI and receiving CKRT.

**Methods:**

Six investigators queried the freely available versions of six LLMs (ChatGPT, Claude, Google Gemini, Microsoft Copilot, OpenEvidence, and Perplexity) from July to September 2025 using three standardized vignettes asking for dosing recommendations and rationale to meet prespecified pharmacodynamic targets: (i) adult with AKI not on dialysis receiving cefepime, (ii) child receiving CKRT and cefepime, and (iii) toddler receiving high‐effluent CKRT and meropenem. To assess inter‐iteration consistency, each investigator also prompted one LLM three times for each vignette. Responses were parsed for concordance with recommendations in primary literature. LLM “reasoning” was evaluated for use of pharmacokinetic (PK) equations, citation accuracy versus confabulation, acknowledgment of uncertainty, and recommendations for therapeutic drug monitoring (TDM) for efficacy or safety.

**Results:**

LLM‐generated dosing recommendations varied widely. Mean concordance with literature‐based recommendations was 63% (range: 28%–94%). LLMs produced variable responses to the same user upon multiple iterations, with variance in daily maintenance doses recommended ranging from 0 to 1000%. OpenEvidence universally cited relevant sources, whereas other LLMs leveraged sources inconsistently or confabulated them. Each recommended TDM, though only Claude consistently acknowledged its own uncertainty.

**Conclusion:**

Freely available LLMs produce highly variable and often discordant antibiotic dosing recommendations for patients with AKI and receiving CKRT. Although valuable for hypothesis generation and literature retrieval, LLM outputs should not be used in isolation for drug dosing in critically ill patients with kidney dysfunction.

## 1 Introduction

Patients with acute kidney injury (AKI) and those receiving continuous kidney replacement therapy (CKRT, formerly known as continuous renal replacement therapy, or CRRT) represent a vulnerable group with a high risk of mortality [1, 2, 3]. These patients frequently have altered drug pharmacokinetics (PK) due to both alterations in physiology from critical illness itself and from the extracorporeal drug device [4, 5]. Despite these PK alterations, there is a paucity of data regarding appropriate drug dosing for many critical medications administered to patients with AKI and those receiving CKRT [5].

Over the past several years, artificial intelligence (AI) has increasingly been studied and utilized to enrich the practice of medicine. Specifically, ChatGPT and other large language models (LLMs), which are computer programs that can generate text resembling human speech, have generated interest in supporting clinicians, given their ease and rapidity of use [6]. Some, such as OpenEvidence, have been trained on medical literature and are marketed specifically to support providers in clinical decision‐making [7]. Existing and proposed applications of LLMs in health care include assistance with clinical documentation and workflow support, augmentation of patient communication and education, extraction of medication safety data from existing reports, and clinical decision support [8, 9]. Existing reviews [6, 8] have highlighted that despite these potential applications, LLMs have serious limitations that must be considered prior to using their output for patient care. These include a lack of optimization for the medical context, non‐comprehensiveness (e.g., providing overly generalized or incomplete recommendations), non‐reproducibility (i.e., receiving different answers to the same prompt given iteratively), the provision of verifiably incorrect information, and inconsistent or incorrect incorporation of citations.

Despite these limitations, given the availability of LLMs to the end‐user, with their rapid generation of responses to inquiries with outputs resembling genuine human communication, clinicians may use LLMs to provide dosing recommendations for patients with kidney dysfunction, including dosing for patients on CKRT. Therefore, we evaluated the potential accuracy and consistency of a subset of freely available LLMs in providing appropriate literature‐based dosing recommendations for patients with kidney dysfunction, including those receiving CKRT.

## 2 Methods

Six LLMs that are freely available to the end‐user were identified: ChatGPT, Claude (Anthropic), Google Gemini, Microsoft Copilot, OpenEvidence, and Perplexity.

Each of the following cases was entered into each LLM (verbatim; abbreviations are defined in the Appendix A):

Prompt 1: I have a 25‐year‐old female 60 kg patient who has sustained Stage 3 acute kidney injury and has been anuric for 24 h; serum creatinine is 3.0 mg/dL, yesterday it was 1.5 mg/dL. What cefepime dosing regimen would you recommend to meet the pharmacodynamic target of 100% *fT* > 1× and 4× MIC for *Pseudomonas* using an MIC of 8 mg/L?

*Then, once the initial response was provided*: Can you explain how you came to this conclusion?

Prompt 2: I have an 11‐year‐old male 35 kg patient receiving CRRT with the PrisMax and an HF1000 filter for acute kidney injury due to complications after bone marrow transplant and is receiving CVVHDF with a total effluent flow of 2000 mL/h/1.73 m^2^. The patient is anuric. What cefepime dosing regimen would you recommend to meet the pharmacodynamic target of 100% *fT* > 1× and 4× MIC for *Pseudomonas* using an MIC of 8 mg/L? *Note: The traditional CRRT nomenclature was specifically used in the LLM prompts over the more recent CKRT to ensure identification and incorporation of as much previously published literature as possible*.

*Then, once the initial response was provided*: Can you explain how you came to this conclusion?

Prompt 3: I have a 2‐year‐old female 10 kg patient receiving CRRT with the PrisMax and an HF20 filter for acute kidney injury and acute hepatic failure and is receiving CVVHD with a total effluent flow of 8000 mL/h/1.73 m^2^ to aid in ammonia clearance. The patient has a BSA of 0.3 m^2^. The patient is still urinating 2 mL/kg/h while on circuit. Given clinical concern for infection, we would like to administer meropenem at adequate doses and frequencies to meet the pharmacodynamic targets of 100% *fT* > 1× and 4× MIC for *Pseudomonas* using an MIC of 2 mg/L. Please use a published population pharmacokinetic model to support your answer.

*Then, once the initial response was provided, can* you explain how you came to this conclusion?

*Then, once the second response was provided*: How would you adjust this if the patient becomes anuric?

Cases were selected based on the investigators' clinical and research expertise and were designed to represent a progression in clinical complexity and evidentiary uncertainty, ranging from a commonly used antimicrobial in a young adult with AKI to pediatric scenarios incorporating smaller body size, variable CKRT intensity, and residual kidney function. To contextualize the responses, we formulated potential “correct” answers based on our own review of the literature on the subject and clinical experience as nephrologists, clinical pharmacologists, intensivists, and pharmacists, along with dosing recommendations available in LexiComp, a commonly used drug reference in hospitals [10]. This consensus was based on group agreement after independent review of the literature and real‐time simultaneous discussion of each case prior to entering the cases into LLMs. Our consensus recommendations are hereafter described as “primary literature” for brevity. We then parsed each response to identify a loading dose, maintenance dose, infusion times, and the dosing interval recommended. Concordance was then assessed for the maintenance dose with the primary literature and with recommendations in LexiComp. Concordance was defined as follows: concordant (same dose, interval, and infusion time), partial (same total daily dose but different interval or infusion time), or discordant (different total daily dose). See Figure 1 for details. Since loading doses are variably reported in both the literature and in LLM outputs, concordance was only assessed with literature recommendations based on maintenance doses. At times, the LLM adjusted the response upon being asked to explain how it came to the conclusion provided. In our summary tables, we have presented only the first response provided by the LLM since this is what would be first presented to a user engaging with the LLM.

**FIGURE 1:**
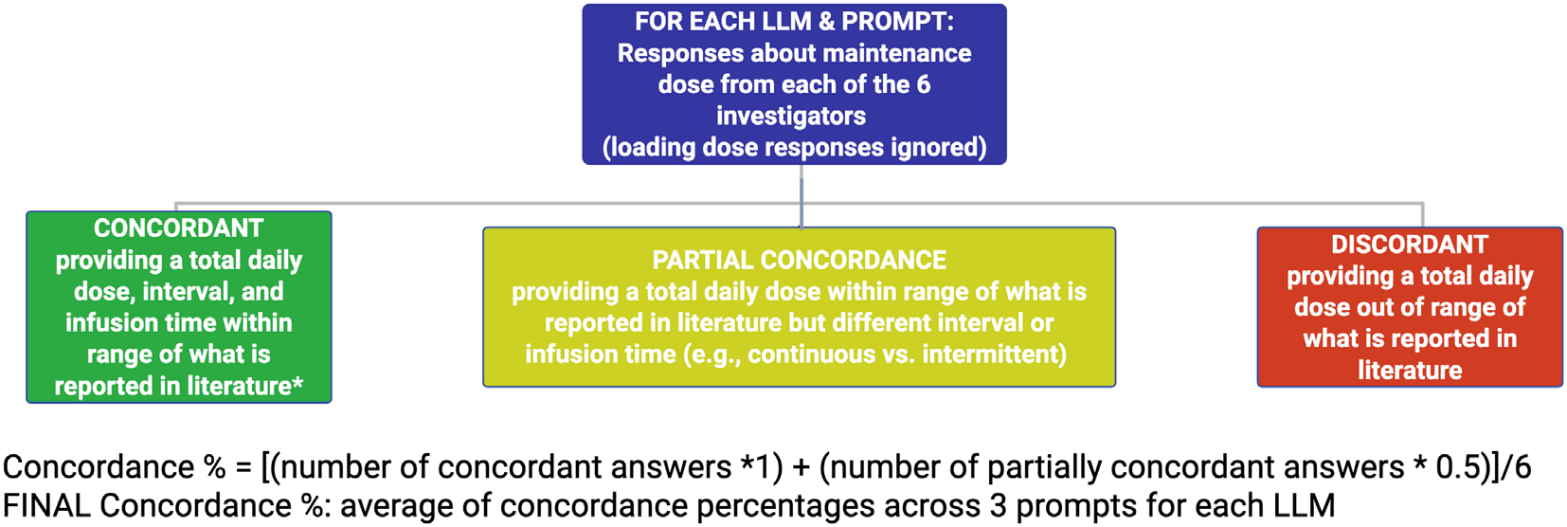
Summary of concordance calculations. LLM, large language model. MIC, minimum inhibitory concentration. *Definition of concordance was agreed by all investigators; consensus was reached about literature recommendations. If separate recommendations given for 1× and 4× MIC each were given half weight to total score. If no response is provided, classified as discordant.

Each of six users entered these prompts between July 22, 2025 and September 30, 2025. We used the freely available (non‐subscription) version of the LLM available at that point in time, except in the cases where the user had an institutional subscription to Microsoft Copilot (Reviewers 2 and 5). Microsoft Copilot and OpenEvidence did not provide information regarding the specific software iteration used to power the AI assistant. For the remaining LLMs, the software used was the GPT‐5 iteration of ChatGPT, Claude 3.5 Sonnet 4 for Claude‐Anthropic, Gemini 2.5 Flash for Google Gemini, and Sonar Llama 3.3 for Perplexity.

In the event that prior use of each LLM influenced the output provided, we asked each of the investigators to characterize their prior experience using each LLM prior to participating in this project. This was categorized as “None” if the investigator had never used the LLM prior to the study, “Minimal” if used fewer than five times, “Moderate” if used 5–15 times, and “Extensive” if used more than 15 times (Table S1).

In addition, we were interested in describing not only the variation in responses provided among users for each LLM but also the variation in responses when one user provided the same prompt to the same LLM multiple times. Therefore, we had each investigator give the same series of prompts to a single LLM three times, closing out of their browser and opening a new browser session in between iterations. Inter‐iteration consistency was categorized as: (i) no consistency (all three responses differed), (ii) partial consistency (two of three responses matched), or (iii) complete consistency (all three responses matched). Finally, if all three iterations provided the same response, this was quantified as three pairs (1–2, 2–3, and 1–3). In addition, we specified the percent variation in the total daily dose recommended in each iteration as a function of the lowest maintenance dose recommended (e.g., variation from 200 to 2000 mg would be defined as 900% variation).

In an effort to describe the “reasoning” each LLM *appeared* to have used to arrive at its dosing recommendation, we reviewed the text output from each LLM to assess a series of predefined criteria. We qualified the response as “yes” if the LLM did any of the following in response to the initial prompt or upon asking for justification:

First, we looked to see whether PK equations were used in the response, and if so, whether the equations used appropriate and logical? PK equations were defined as equations relating patient information to a primary or secondary PK parameter such as clearance, volume of distribution, or half‐life; equations simply regarding kidney function (e.g., estimated glomerular filtration rate), CKRT parameters (e.g., effluent flow rate), or dosing calculations (e.g., multiplying weight by a weight‐based dose) were not considered PK equations.

Second, given the potential risk of LLMs confabulating sources that do not exist, we identified if primary literature sources were cited. If sources were cited, we investigated if the sources mentioned could be traced to real articles or if the articles were confabulated by the LLM, if the sources were tailored to the patient's age, and if the sources were tailored to the patient's condition (i.e., AKI and/or CKRT depending on the prompt).

Third, given that LLM output may give the *prima facie* appearance of truth even when it is incorrect or generated based on flawed assumptions, we identified if uncertainty in the LLM's ability to answer the question was acknowledged in the response.

Fourth, given the uncertainty and inter‐individual variability in beta‐lactam exposure in these populations, we identified if therapeutic drug monitoring (TDM) was recommended in the LLM's response.

Fifth, since our prompts asked for the LLM to comment on drug regimens that would achieve stringent pharmacodynamic targets, we looked to see if the LLM explicitly commented on the amount of time the plasma drug concentration exceeded the minimum inhibitory concentration (T > MIC).

Finally, since there are concerns regarding neurotoxicity with excessive exposure to beta‐lactams, particularly cefepime, we identified if the LLM mentioned neurotoxicity in the response, regardless of how toxicity‐associated drug exposure was quantified (maximum concentration or C_max_, minimum concentration or C_min_, area under the curve of the concentration‐time profile or AUC, etc.).

## 3 Results

The dose recommended for each patient scenario for each user is provided in Table S2. Responses varied markedly from one user to the next regarding total daily dose, interval, and infusion time. Concordance of the recommendations from primary literature ranged widely: 94.3% for OpenEvidence, 69.3% for Copilot, Claude, and Perplexity, 50% for ChatGPT, and 27.9% for Gemini. Concordance with recommendations in Lexicomp followed a similar pattern, with 72.3% for OpenEvidence, 69.3% for Copilot, 63.7% for Perplexity, 41.7% for ChatGPT, 27.7% for Claude, and 5.7% for Gemini.

The dose recommended by individual users giving the same prompt across multiple iterations to a given LLM is presented in Table S3. Responses often varied significantly between iterations. The mean number of pairs of identical recommendations across the three iterations of each prompt was 2.25 for OpenEvidence, 1.5 for Perplexity and Copilot, 0.75 for Gemini, 0.5 for ChatGPT, and 0.25 for Claude. The mean percent variation in the maintenance daily dose was 50% for OpenEvidence, 108% for Claude (though Claude occasionally declined to provide a recommendation, which is not reflected in this percentage), 126% for Copilot, 248% for Gemini, 263% for Perplexity, and 469% for ChatGPT.

A summary of the “reasoning” provided by each LLM is shown in Table 1. Most LLMs used PK equations in many of their responses, with ChatGPT using them most frequently (83% of the time), though OpenEvidence and Perplexity never used them. When present, PK equations were typically appropriate for the question at hand (95% of cases), though equations were often simply straightforward PK equations regarding volume of distribution and half‐life, and there were occasional errors in arithmetic. All LLMs cited sources at least some of the time, with OpenEvidence citing them without exception and Claude and Gemini only citing them in 33% of cases. The sources provided by OpenEvidence and Perplexity were uniformly real and appropriately tailored to the patient's weight and medical condition, whereas one third of the sources provided by Gemini and approximately one sixth of those provided by Claude were confabulated. Claude was consistent in reporting its own uncertainty in answering the questions; however, across LLMs, uncertainty was acknowledged only 37% of the time on average. Virtually all (98%) responses recommended TDM. All responses acknowledged the therapeutic target of maximizing T > MIC; toxicity was mentioned frequently, in 88% of responses.

**TABLE 1:** Evaluation of justifications given for the answers provided by each LLM.

Review criteria	ChatGPT (%)	Claude – Anthropic (%)	Google Gemini (%)	Microsoft Copilot (%)	Open evidence (%)	Perplexity (%)
Were PK equations used?	15/18 (83)	10/18 (56)	11/18 (61)	6/18 (33)	0/18 (0)	0/18 (0)
If Y: Were PK equations appropriate and logical?	15/15 (100)	9/10 (90)	10/11 (91)	6/6 (100)	NA	NA
Were sources cited?	14/18 (78)	6/18 (33)	6/18 (33)	14/18 (78)	18/18 (100)	15/18 (83)
If Y: Were sources real?	14/14 (100)^a^	5/6 (83)	2/3 (67)^b^	14/14 (100)	18/18 (100)	15/15 (100)
If Y: Were (real) sources tailored to patient age?	10/14 (71)	5/5 (100)	1/2 (50)	12/14 (86)	18/18 (100)	15/15 (100)
If Y: Were (real) sources tailored to patient condition (AKI, dialysis)?	14/14 (100)	5/5 (100)	2/2 (100)	12/14 (86)	18/18 (100)	15/15 (100)
Was uncertainty in the LLM's ability to answer the question acknowledged?	0/18 (0)	18/18 (100)	6/18 (33)	1/18 (6)	9/18 (50)	6/18 (33)
Was TDM recommended?	18/18 (100)	18/18 (100)	18/18 (100)	16/18 (89)	18/18 (100)	18/18 (100)
Was efficacy (T > MIC) mentioned?	18/18 (100)	18/18 (100)	18/18 (100)	18/18 (100)	18/18 (100)	18/18 (100)
Was toxicity mentioned?	16/18 (89)	10/18 (56)	18/18 (100)	16/18 (89)	18/18 (100)	17/18 (94)

Abbreviations: AKI, acute kidney injury; LLM, large language model; PK, pharmacokinetic; T > MIC, time concentrations exceed bacterial minimum inhibitory concentration; TDM, therapeutic drug monitoring; Y, yes.

^a^ In one case, ChatGPT cited a real source but the in‐line citation misattributed the reference.

^b^ In three of the six sources for Google Gemini, the copy‐pasted version of the LLM output had superscripts but no links to individual sources, so the veracity and applicability of these sources could not be individually verified.

## 4 Discussion

In this study, we evaluated the accuracy, consistency, and reasoning processes of freely available LLMs when asked to generate antibiotic dosing recommendations for patients in three cases of hypothetical patients with AKI and those receiving CKRT. We found substantial variability in LLM‐generated recommendations, frequent discordance with primary literature, and marked inconsistency both across users and within repeated iterations of identical prompts. Together, these results underscore important limitations of LLMs when applied to medication dosing in critically ill patients with kidney dysfunction, a domain in which precision, transparency, and physiological grounding are vital.

Drug dosing in AKI and CKRT is inherently complex. Antibiotic clearance reflects not only residual kidney function, but also CKRT modality, effluent rate, filter characteristics, hepatic function, protein binding, volume of distribution, and rapid changes in fluid balance and perfusion. Even among experts, dosing recommendations often involve ranges rather than a single “correct” answer. However, the degree of variation observed here, with daily maintenance doses differing by up to 1000% for the same clinical vignette, far exceeds what would be considered clinically acceptable.

Importantly, the inconsistency seen in these cases was not confined to differences between LLM platforms. Repeated iterations of the same prompt to the same model yielded materially different recommendations, reflecting an inherent limitation of the outputs of probabilistic language generation. Although having non‐deterministic output may be advantageous in creative or exploratory tasks, it is fundamentally misaligned with clinical scenarios in which users rely on reproducible, defensible guidance. This concern is amplified when LLM outputs are presented in confident, authoritative language, potentially obscuring underlying uncertainty, and may be misinterpreted as the single “correct” answer by early learners when experts may recognize that ranges of appropriate dosing regimens may exist.

Although LLMs often explicitly employed PK equations or mechanistic reasoning to justify dosing recommendations, the underlying assumptions built into the equations used and the outputs were not always internally consistent. For example, in one iteration, Claude, which used PK equations in approximately half of its responses, used math correctly to determine that a body surface area (BSA)‐normalized CKRT clearance of 8000 mL/h/1.73 m^2^ would correspond to a total clearance of around 1390 mL/h for a child with a BSA of 0.3 m^2^, and used basic first‐order PK equations to determine expected clearance, volume of distribution, and half‐life from an existing meropenem population PK model. However, Claude then recommended a maintenance dose of 320–400 mg/day as a continuous infusion without specifying precisely how it arrived at this dose, incorrectly reporting that this would correspond to 13–17 mg/kg/h (for a 10‐kg child, this would correspond to 32–40 mg/kg/day or 1.3–1.7 mg/kg/h). This output is wrong in two ways: the total daily dose is well below the literature consensus of 80–120 mg/kg/day as a continuous infusion, *and* the math relating mg/day to mg/kg/h is incorrect. Yet, to an end user looking quickly, the appearance of equations may appear quite convincing unless the mathematical equations are critically parsed. This appearance of mechanistic rigor may be more dangerous than providing no answer at all, as it can mislead users into trusting incorrect recommendations. Consequently, LLM output must be reviewed by a user with expertise in the field and a discerning eye for errors and inconsistencies prior to use in direct patient care.

Equally concerning was the inconsistent use of references. With the exception of OpenEvidence, which was explicitly trained on medical literature, and Perplexity, which both consistently cited relevant literature, many LLMs either cited sources inaccurately, referenced irrelevant guidelines, or generated citations that could not be verified. In a high‐risk setting such as antibiotic dosing in AKI or CKRT, citation confabulation is not a trivial flaw: it directly undermines the trustworthiness of the recommendation and may falsely reassure users that guidance is evidence‐based when it is not.

Several prompts in our study intentionally incorporated clinical ambiguity (e.g., an anuric patient with AKI whose likelihood of kidney recovery was uncertain). Yet, most LLM responses failed to acknowledge how such uncertainty should influence dosing decisions, nor did they universally discuss dynamic reassessment as kidney function evolves. Claude was a notable exception in consistently acknowledging uncertainty, but even here this did not reliably translate into more concordant dosing recommendations.

To better contextualize these limitations, it is essential to understand that LLMs are trained predominantly on population‐level data and static text [11]. More specifically, they are so‐called autoregressive “neural networks” that are trained to estimate conditional probability distributions over sequences of “tokens”, generating responses by sampling from these distributions rather than executing truly rule‐based logic or mechanistic calculations [12]. Their behavior is shaped by pre‐training on enormous volumes of text followed by reinforcement learning from human feedback (RLHF), which prioritizes fluency, coherence, and perceived helpfulness over internal consistency or numerical correctness [13]. That is, LLMs generate outputs by probabilistic token prediction rather than executing mechanistic or rule‐based calculations. As a result, they approximate dosing recommendations through pattern recognition rather than applying PK principles. As a result, unless explicitly constrained, LLMs do not perform PK calculations or apply mass‐balance principles, but instead approximate dosing recommendations by pattern‐matching to textual examples in the training data.

This probabilistic sampling framework introduces inherent non‐determinism, such that repeated queries—even with identical prompts—can yield meaningfully different outputs, particularly for tasks without a single dominant textual “answer,” as is typical for drug dosing in AKI and CKRT. That is, LLMs do not possess an internal representation of physiology or PK systems; they do not “solve” dosing problems using clearance equations, mass balance, or time‐dependent models, but instead infer plausible‐sounding recommendations from textual associations in the training data. The extreme inter‐iteration variability observed in our study, including maintenance dose variation exceeding 400% for some models, is therefore not an anomaly but a predictable consequence of model design.

In high‐stakes settings where correct dosing depends on integrating patient‐specific parameters into mechanistic models, these features predispose LLMs to generate confident but internally inconsistent recommendations that may not be anchored in pharmacologic first principles. As such, they struggle to incorporate longitudinal changes or broader clinical context, including comorbidities or concurrent medications that could meaningfully alter antibiotic PK and toxicity risk. To be explicit, the administration of sub‐therapeutic doses can result in inadequate antimicrobial effect and treatment failure, while supratherapeutic doses of beta‐lactams, particularly cefepime, can result in neurotoxicity [14]. The recommendation of potentially supratherapeutic or toxic doses in some AKI scenarios (e.g., Microsoft Copilot twice recommending a maintenance dose of 4000–6000 mg of cefepime per day in an anuric patient) in our study highlights the danger of applying these probability‐based outputs to individuals without explicit safeguards.

Importantly, the growing accessibility of LLMs raises important ethical and medico‐legal questions. When an AI‐generated recommendation contributes to patient harm, responsibility remains with the clinician, but the opacity and authority of LLM outputs may complicate clinical decision‐making and informed consent. At present, there is no standardized regulatory framework governing the clinical use of general‐purpose LLMs for medication dosing, nor clear guidance regarding liability.

Additionally, practical considerations such as cost, computing infrastructure, and institutional oversight remain unresolved. Although freely available models lower barriers to access, they also bypass traditional mechanisms of validation, version control, and quality assurance that are expected for clinical decision support tools [15].

Our findings do not suggest that LLMs lack value in clinical pharmacology. On the contrary, many responses demonstrated utility in hypothesis generation, synthesis of general principles, and retrieval of relevant literature. Used judiciously, these tools may enhance clinician efficiency and support education [16]. In accordance, it is notable that a 2025 survey of approximately 1400 practicing clinical pharmacists found that although approximately one‐third had an inherent distrust of AI, approximately two‐thirds felt that AI could enhance their professional effectiveness and productivity [17]. Regardless, our results clearly indicate that LLM‐generated dosing recommendations should not be used in isolation for direct patient care in AKI or CKRT.

Moreover, discordance in this study should not be interpreted solely as disagreement between LLM‐generated responses and our review of the primary literature. Our adjudicated reference standard reflected an interdisciplinary synthesis of primary literature and the applied clinical expertise of nephrologists, clinical pharmacologists, an intensivist, and clinical pharmacists. This distinction is clinically relevant because many of the questions posed to the LLMs mirror real‐world scenarios in which an immediately actionable answer may be sought before a clinician has the time, literature access, or specialist support needed to perform a comprehensive review. Indeed, the appeal of LLMs is partly driven by this gap: they can generate a coherent response within minutes, whereas expert synthesis may require substantially more time, access to subscription resources, and multidisciplinary interpretation. Thus, our findings should not be taken to imply that LLMs were compared against what any individual clinician could reproduce at the bedside under routine time constraints. Rather, we intentionally evaluated whether current LLMs could approximate a high‐quality, expert‐informed answer in complex nephrology and pharmacotherapy scenarios. The observed discordance therefore highlights both the promise and the risk of LLM use: these tools may be most attractive in settings with limited access to nephrology or clinical pharmacy expertise, but those same settings may also be least equipped to recognize subtle inaccuracies, omissions, or overconfident reasoning.

A more promising future direction may involve leveraging LLMs to improve the accessibility and usability of curated, authoritative resources, such as primary PK studies, dosing compendia, and institutional guidelines, rather than asking them to generate *de novo* recommendations. Integrating transparent assumptions, mechanistic PK models, and real‐time patient data within regulated clinical decision support systems may ultimately offer safer and more reliable outcomes [18].

Our study has several limitations. First, concordance was assessed against our interpretation of literature‐based criteria that themselves reflect ongoing debate and heterogeneity in clinical practice. Exact percentages of concordance may therefore vary depending on how “correctness” is defined. That said, even under conservative definitions, substantial differences across models persisted. Second, prompts necessarily simplified complex clinical scenarios, and we did not evaluate paid or enterprise versions of these platforms, which may differ in performance. Third, for this exploratory study, we only queried three clinical scenarios. Future studies could strengthen this analysis by querying additional iterations more systematically (e.g., each possible combination of adult versus child, AKI with or without CKRT, CKRT with high versus standard effluent flows, and CKRT with minimal versus substantial residual urine output). Fourth, the prompts we provided were intentionally quite specific to encourage LLMs to provide answers that we could compare with specificity against existing published literature; however, this is likely not generalizable to the use of an LLM by a user with less experience in pharmacology who may ask for recommendations using a prompt with fewer details and less specificity. Fifth, in many cases, an organism and/or MIC are not known and dosing must be performed empirically, so such precise vignettes may not be applicable in many clinical settings. Sixth, the inclusion of users who had a variety of experience with using individual LLMs and some users who had institutional subscriptions diluted the internal validity of this study; that said, since real‐life users of LLMs have varying combinations of subscriptions and prior personal use of each LLM when engaging with them, we feel that the “real‐world” nature of this heterogeneity contributes to the generalizability of the findings. Seventh, this study should be interpreted as a descriptive case series rather than a hypothesis‐testing analysis. Because only three cases were formulated, formal statistical analysis could not be conducted, and the findings should be viewed as exploratory and hypothesis‐generating. A larger number of cases with LLM users from more sites will be needed to determine whether the patterns observed here are reproducible and to support more definitive conclusions. Finally, our findings represent a snapshot in time; LLM behavior is known to evolve over time, sometimes in unpredictable ways, including changes in consistency across iterations. It is probable that a study conducted using the same prompts 1, 2, or 3 years after this study was performed could result in meaningfully different outcomes, so AI performance should continue to be assessed longitudinally and iteratively over time.

## 5 Conclusions

When using three prompts, freely available LLMs generated antibiotic dosing recommendations for patients with AKI and receiving CKRT that were highly variable, inconsistently reasoned, and often discordant with consensus review of existing data in peer‐reviewed literature and a tertiary reference. The combination of confident presentation, limited PK‐based reasoning, citation confabulation, and non‐reproducibility poses meaningful risks in high‐stakes clinical settings. Until these limitations are addressed, LLMs should be viewed as adjunctive tools for exploration and education, not as substitutes for expert judgment, mechanistic reasoning, or evidence‐based dosing strategies in critically ill patients with kidney dysfunction.

## Author Contributions

**H. Rhodes Hambrick:** conceptualization, data curation, formal analysis, writing – original draft, methodology, project administration, writing – review and editing, investigation. **Samuel Dubinsky:** conceptualization, methodology, data curation, writing – review and editing, investigation. **Danica Quickfall:** conceptualization, methodology, data curation, writing – review and editing, investigation. **Shannon Reinert:** conceptualization, methodology, data curation, writing – review and editing, investigation. **Sonya Tang Girdwood:** conceptualization, supervision, data curation, methodology, investigation, writing – review and editing. **Gideon Stitt:** conceptualization, methodology, supervision, writing – review and editing, data curation, investigation.

## Funding

The authors have nothing to report.

## Conflicts of Interest

The authors declare no conflicts of interest.

## Supporting information


**Table S1:** Experience using LLMs prior to starting the project (as of 7/22/25).
**Table S2:** Summary of the first dosing recommendation provided from each LLM when prompted by each user compared with recommendations available from a consensus review of the primary literature and in LexiComp. Concordance with the primary literature and LexiComp was classified as concordant, partial (if the daily dose was the same but the infusion time or interval differed), or discordant (if the recommendation differed in total daily dose recommended).
**Table S3:** Evaluation of consistency across iterations for a given user.

## Data Availability

The data that support the findings of this study are available from the corresponding author upon reasonable request.

## Appendix A

Abbreviations used in LLM prompts

- 100% *fT* > 1× MIC—free concentrations exceeding one time the minimum inhibitory concentration for 100% of the dosing interval
- 100% *fT* > 4× MIC—free concentrations exceeding four times the minimum inhibitory concentration for 100% of the dosing interval
- BSA—body surface area.
- CRRT—continuous renal replacement therapy.
- CVVHD—continuous veno‐venous hemodialysis
- CVVHDF—continuous veno‐venous hemodiafiltration
