# Supplementary material for: User Beware: Inaccuracy and Inconsistency of Large Language Models in Providing Precision Dosing Recommendations for Patients With Kidney Impairment—A Case Series
Source: Pharmacotherapy. 2026 Oct 1;46(10):e70207. doi: 10.1002/phar.70207 (PMC13628145; doi:10.1002/phar.70207)
Supplement: Supplementary file 1 [file PHAR-46-0-s001.docx]

**Supplementary Information**

**Supplemental Table 1**: Experience Using LLM prior to starting project (as of 7/22/25)

|  | **User 1** | **User 2** | **User 3** | **User 4** | **User 5** | **User 6** |
| --- | --- | --- | --- | --- | --- | --- |
| **ChatGPT** | Extensive | Minimal | Extensive | Minimal | Extensive | Extensive |
| **Claude - Anthropic** | None | None | None | None | None | Moderate |
| **Google Gemini** | Extensive | Extensive | Extensive | Extensive | Extensive | None |
| **Microsoft CoPilot** | None | Minimal | Extensive | None | None | Moderate |
| **Open Evidence** | Minimal | None | None | None | None | None |
| **Perplexity** | None | None | None | None | Moderate | None |

None: never used before, Minimal: have used <5 times, Moderate: have used 5-15 times, Extensive: have used >15 times

**Supplementary Table 2**. Summary of first dosing recommendation provided from each LLM when prompted by each user compared with recommendations available from consensus review of primary literature and in LexiComp. Concordance with primary literature and LexiComp was classified as concordant, partial (if daily dose the same but infusion time or interval differed), or discordant (if recommendation differed in total daily dose recommended).

|  | | **Prompt 1**: 25 y.o. 60 kg female with stage 3 acute kidney injury, anuric x 24 hours with serum creatinine of 3.0 mg/dL, recommended cefepime dosing regimen to meet target of 100% *f*T>1x and *f*T>4x MIC for *Pseudomonas* assuming an MIC of 8 mg/dL | | | | | | | | | | | | | | | |
| --- | --- | --- | --- | --- | --- | --- | --- | --- | --- | --- | --- | --- | --- | --- | --- | --- | --- |
| Literature Recommendation^1^ | | For 1x MIC: 1000 mg every 24 hours or 500 mg every 12 hours as a 30-minute infusion  For 4x MIC: Increase to 2000 mg every 24 hours or 1000 mg every 12 hours as a 30-minute infusion  Consider extended or continuous infusions to minimize peaks while maximizing T > MIC  If no recovery of kidney function, may need to reduce dose/frequency further | | | | | | | | | | | | | | | |
| Lexicomp Recommendation^2^ | | 2000 mg every 24 hours or 1000 mg every 12 hours as a 30-minute infusion, prefer the latter to maximize T > MIC | | | | | | | | | | | | | | | |
| **LLM** | | **User 1** | | **User 2** | | **User 3** | | | **User 4** | | | **User 5** | | | **User 6** | | |
| **ChatGPT**  Average Literature Concordance:   - PL: 50% - LC: 12.5% | | LD: 2000 mg  T_inf_: 30-60 min  MD: 1000 mg  T_inf_: 3-4 hr  Int: 24 hr  PL: Concordant  LC: Discordant | | LD: 2000 mg  T_inf_: 30-60 min  MD: 1000 mg  T_inf_: N.S.; 3-4 hr if 4x MIC  Int: 24-48 hr  PL: Concordant  LC: Discordant | | LD: 2000 mg  T_inf_: N.S.  MD: 3000 - 4000 mg  T_inf_: 24 hr  Int: 24 hr  PL: Discordant  LC: Discordant | | | LD: 2000 mg  T_inf_: 3-4 hr  MD: None until clearance resumes  PL: Discordant  LC: Discordant | | | LD: 2000 mg  T_inf_: N.S.  MD: 2000 mg  T_inf_: 3-4 hr  Int: 24 hr  PL: Concordant  LC: Partial | | | LD: 2000 mg  T_inf_: 3-4 hr  MD: None until clearance resumes  PL: Discordant  LC: Discordant | | |
| **Claude - Anthropic**  Average Literature Concordance:   - PL: 100% - LC: 25% | | LD: 2000 mg  T_inf_: N.S.  MD: 1000 mg  T_inf_: N.S.  Int: 24 hr  PL: Concordant  LC: Discordant | | LD: 2000 mg  T_inf_: N.S.  MD: 1000 mg  T_inf_: N.S.  Int: 24 hr  PL: Concordant  LC: Discordant | | LD: N.S.  T_inf_: N.S.  MD: 1000 mg  T_inf_: 4 hr  Int: 12 hr  PL: Concordant  LC: Partial | | | 1x MIC \| 4x MIC  LD: N.S. \| N.S.  T_inf_: N.S. \| N.S.  MD: 1000 mg \| 2000 mg  T_inf_: 4 hr \| 4 hr  Int: 24 hr \| 24 hr  PL: Concordant \| Concordant  LC: Discordant \| Partial | | | LD: 2000 mg  T_inf_: N.S.  MD: 1000 mg  T_inf_: 3-4 hr  Int: 12 hr  PL: Concordant  LC: Partial | | | LD: 200 mg  T_inf_: N.S.  MD: 1000 mg  T_inf_: N.S.  Int: 24 hr  PL: Concordant  LC: Discordant | | |
| **Google Gemini**  Average Literature Concordance:   - PL: 66.7% - LC: 0% | | LD: N.S.  T_inf_: N.S.  MD: 480 mg  T_inf_: 24 hr  Int: 24 hr  PL: Discordant  LC: Discordant | | LD: 2000 mg  T_inf_: 30 min  MD: 1000 mg  T_inf_: 3 - 4 hr  Int: 24 hr  PL: Concordant  LC: Discordant | | LD: 2000 mg  T_inf_: 30 min  MD: 1500 mg  T_inf_: 24 hr  Int: 24 hr  PL: Concordant  LC: Discordant | | | N/A, “Recommend consultation with specialists” | | | LD: 1000 mg  T_inf_: 30 min  MD: 1500 mg  T_inf_: 24 hr  Int: 24 hr  PL: Concordant  LC: Discordant | | | LD: 2000 mg  T_inf_: 30-60 min  MD: 1000 mg  T_inf_: 24 hr  Int: 24 hr  PL: Concordant  LC: Discordant | | |
| **Microsoft CoPilot**  Average Literature Concordance:   - PL: 33.3% - LC: 8.3% | | LD: 2000 mg  T_inf_: N.S.  MD: 2000 mg  T_inf_: 3-4 hr  Int: 8 hr  PL: Discordant  LC: Discordant | | LD: 2000 mg  T_inf_: 30-60 min  MD: 1000 - 2000 mg  T_inf_: 24 hr  Int: 24 hr  PL: Concordant  LC: Partial | | LD: N.S.  T_inf_: N.S.  MD: 2000 mg  T_inf_: 4 hr  Int: 8 hr  PL: Discordant  LC: Discordant | | | LD: 2000 mg  T_inf_: N.S.  MD: 4000 - 6000 mg  T_inf_: 24 hr  Int: 24 hr  PL: Discordant  LC: Discordant | | | LD: 2000 mg  T_inf_: N.S.  MD: 1000 mg  T_inf_: 4 hr  Int: 24 - 48 hr  PL: Concordant  LC: Discordant | | | LD: 2000 mg  T_inf_: 30 min  MD: 4000 - 6000 mg  T_inf_: 24 hr  Int: 24 hr  PL: Discordant  LC: Discordant | | |
| **Open**  **Evidence**  Average Literature Concordance:   - PL: 83% - LC: 17% | | LD: N.S.  T_inf_: N.S.  MD: 1000 mg  T_inf_: N.S.  Int: 24 hr  PL: Concordant  LC: Discordant | | LD: N.S.  T_inf_: N.S.  MD: 1000-2000 mg  T_inf_: 24 hr  Int: 24 hr  PL: Concordant  LC: Partial | | LD: N.S.  T_inf_: N.S.  MD: 1000-2000 mg  T_inf_: 24 hr  Int: 24 hr  PL: Concordant  LC: Partial | | | LD: N.S.  T_inf_: N.S.  MD: 3000 mg  T_inf_: 24 hr  Int: 24 hr  PL: Discordant  LC: Discordant | | | LD: N.S.  T_inf_: N.S.  MD: 1000 mg  T_inf_: N.S.  Int: 24 hr  PL: Concordant  LC: Discordant | | | LD: N.S.  T_inf_: N.S.  MD: 1000 mg  T_inf_: N.S.  Int: 24 hr  PL: Concordant  LC: Discordant | | |
| **Perplexity**  Average Literature Concordance:   - PL: 67% - LC: 8% | | LD: 2000 mg  T_inf_: N.S.  MD: 1000 mg  T_inf_: 24 hr  Int: 24 hr  PL: Concordant  LC: Discordant | | LD: N.S.  T_inf_: N.S.  MD: 1000 mg  T_inf_: N.S.  Int: 24 hr  PL: Concordant  LC: Discordant | | LD: 2000-4000 mg  T_inf_: N.S.  MD: 4000-6000 mg  T_inf_: 24 hr  Int: 24 hr  PL: Discordant  LC: Discordant | | | LD: N.S.  T_inf_: N.S.  MD: 1000 mg  T_inf_: 4 hr  Int: 12 hr  PL: Concordant  LC: Partial | | | LD: 1000-2000 mg  T_inf_: N.S.  MD: 500-1000 mg  T_inf_: N.S.  Int: 24 hr  PL: Concordant  LC: Discordant | | | LD: 2000 mg  T_inf_: N.S.  MD: 1500 - 1750 mg  T_inf_: N.S.  Int: 8 hr  PL: Discordant  LC: Discordant | | |
|  | | **Prompt 2**: 11-year-old male anuric 35 kg patient on CRRT with an HF1000 filter for acute kidney injury due to complications after bone marrow transplant and is receiving CVVHDF with a total effluent flow of 2000 mL/hr/1.73 m^2^, recommended cefepime dosing regimen to meet target of 100% *f*T>1x and *f*T>4x MIC for *Pseudomonas* assuming an MIC of 8 mg/dL | | | | | | | | | | | | | | | |
| Literature Recommendation^3^ | For 1x MIC: 50 mg/kg (1750 mg) every 8h as a 30 min infusion, consider 4h infusion to maximize probability of target attainment  For 4x MIC: 150 mg/kg/day (5250 mg/day) as a continuous infusion | | | | | | | | | | | | | | | |  |
| Lexicomp Recommendation^2^ | 50 mg/kg/dose every 8 to 12 hr as a 30 min infusion, consider extended infusions or more frequent administration for critical illness or deep-seated infections | | | | | | | | | | | | | | | |  |
| **LLM** | **User 1** | | **User 2** | | **User 3** | | | **User 4** | | | **User 5** | | | **User 6** | | |  |
| **ChatGPT**  Average Literature Concordance:   - PL: 50% - LC: 50% | LD: 50 mg/kg  T_inf_: 30 min  MD: 42 - 57 mg/kg  T_inf_: 24 hr  Int: 24 hr  PL: Discordant  LC: Discordant | | LD: 50 mg/kg  T_inf_: N.S.  MD: 1750 mg  T_inf_: 3-4 hr  Int: 8 hr  PL: Concordant  LC: Concordant | | LD: 50 mg/kg  T_inf_: N.S.  MD: 100-114 mg/kg  T_inf_: 24 hr  Int: 24 hr  PL: Discordant  LC: Discordant | | | LD: 50 mg/kg  T_inf_: 30 min  MD: 42 - 57 mg/kg  T_inf_: 24 hr  Int: 24 hr  PL: Discordant  LC: Discordant | | | LD: 57 mg/kg  T_inf_: 30 min  MD: 50 mg/kg  T_inf_: 3-4 hr  Int: 8 hr  PL: Concordant  LC: Concordant | | | LD: 50 mg/kg  T_inf_: 3-4 hr  MD: 50 mg/kg  T_inf_: 3-4 hr  Int: 8 hr  PL: Concordant  LC: Concordant | | |  |
| **Claude - Anthropic**  Average Literature Concordance:   - PL: 83% - LC: 0% | LD: 50 mg/kg  T_inf_: 30 min  MD: 50 mg/kg  T_inf_: N.S.  Int: 8 hr  PL: Concordant  LC: Concordant | | LD: 50 mg/kg  T_inf_: “rapid”  MD: 50 mg/kg  T_inf_: N.S.  Int: 8 hr  PL: Concordant  LC: Concordant | | LD: N.S.  T_inf_: N.S.  MD: 50 mg/kg  T_inf_: 3-4 hr  Int: 12 hr  PL: Discordant  LC: Concordant | | | LD: N.S.  T_inf_: N.S.  MD: 50 mg/kg  T_inf_: 3-4 hr  Int: 8 hr  PL: Concordant  LC: Concordant | | | LD: 50 mg/kg  T_inf_: 30 min  MD: 50 mg/kg  T_inf_: 3-4 hr  Int: 8 hr  PL: Concordant  LC: Concordant | | | LD: 50 mg/kg  T_inf_: N.S.  MD: 50 mg/kg  T_inf_: N.S.  Int: 8 hr  PL: Concordant  LC: Concordant | | |  |
| **Google Gemini**  Average Literature Concordance:   - PL: 0% - LC: 0% | LD: 50 mg/kg  T_inf_: 30 min  MD: 28.6 mg/kg  T_inf_: 24 hr  Int: 24 hr  PL: Discordant  LC: Discordant | | LD: 1750 mg  T_inf_: 30 min  MD: 48 mg/kg  T_inf_: 24 hr  Int: 24 hr  PL: Discordant  LC: Discordant | | LD: 500 mg  T_inf_: 30-60 min  MD: 38.6 mg/kg  T_inf_: 24 hr  Int: 24 hr  PL: Discordant  LC: Discordant | | | Recommended consultation with specialists | | | LD: 29 mg/kg  T_inf_: N.S.  MD: 57 mg/kg  T_inf_: 24 hr  Int: 24 hr  PL: Discordant  LC: Discordant | | | LD: 50 mg/kg  T_inf_: 30 min  MD: 50 mg/kg  T_inf_: 24 hr  Int: 24 hr  PL: Discordant  LC: Discordant | | |  |
| **Microsoft CoPilot**  Average Literature Concordance:   - PL: 100% - LC: 100% | LD: 57 mg/kg  T_inf_: 30 min  MD: 42-50 mg/kg  T_inf_: 3-4 hr  Int: 8 hr  PL: Concordant  LC: Concordant | | LD: 50 mg/kg  T_inf_: 30-60 min  MD: 150 mg/kg  T_inf_: 24 hr  Int: 24 hr  PL: Concordant  LC: Concordant | | LD: 57 mg/kg  T_inf_: 30 min  MD: 150 mg/kg  T_inf_: 24 hr  Int: 24 hr  PL: Concordant  LC: Concordant | | | LD: 50 mg/kg  T_inf_: 30-60 min  MD: 150 mg/kg  T_inf_: 24 hr  Int: 24 hr  PL: Concordant  LC: Concordant | | | 1x MIC \| 4x MIC  LD: 50 mg/kg \| 50 mg/kg  T_inf_: 30 min \| 30 min  MD: 50 mg/kg \| 128-142 mg/kg  T_inf_: 3-4 hr \| 24 hr  Int: 8 hr \| 24 hr  PL: Concordant \| Concordant  LC: Concordant \| Concordant | | | LD: 50 mg/kg  T_inf_: 30 min  MD: 150 mg/kg  T_inf_: 24 hr  Int: 24 hr  PL: Concordant  LC: Concordant | | |  |
| **Open Evidence**  Average Literature Concordance:   - PL: 100% - LC: 100% | LD: 50 mg/kg  T_inf_: N.S.  MD: 50 mg/kg  T_inf_: 3-4 hr  Int: 8 hr  PL: Concordant  LC: Concordant | | LD: N.S.  T_inf_: N.S.  MD: 50 mg/kg  T_inf_: 3-4 hr  Int: 8 hr  PL: Concordant  LC: Concordant | | LD: N.S.  T_inf_: N.S.  MD: 50 mg/kg  T_inf_: 3-4 hr  Int: 8 hr  PL: Concordant  LC: Concordant | | | LD: N.S.  T_inf_: N.S.  MD: 50 mg/kg  T_inf_: 3-4 hr  Int: 8 hr  PL: Concordant  LC: Concordant | | | LD: N.S.  T_inf_: N.S.  MD: 50 mg/kg  T_inf_: N.S.  Int: 8 hr  PL: Concordant  LC: Concordant | | | LD: N.S.  T_inf_: N.S.  MD: 50 mg/kg  T_inf_: 3-4 hr  Int: 8 hr  PL: Concordant  LC: Concordant | | |  |
| **Perplexity**  Average Literature Concordance:   - PL: 83% - LC: 83% | LD: 50 mg/kg  T_inf_: 30 min  MD: 50 mg/kg  T_inf_: 3-4 hr  Int: 8 hr  PL: Concordant  LC: Concordant | | LD: N.S.  T_inf_: N.S.  MD: 50 mg/kg  T_inf_: 3-4 hr  Int: 8 hr  PL: Concordant  LC: Concordant | | LD: 50 mg/kg  T_inf_: 30-60 min  MD: 50 mg/kg  T_inf_: 3-4 hr  Int: 8 hr  PL: Concordant  LC: Concordant | | | LD: N.S.  T_inf_: N.S.  MD: 60 mg/kg  T_inf_: 4 hr  Int: 8 hr  PL: Discordant  LC: Discordant | | | LD: N.S.  T_inf_: N.S.  MD: 50 mg/kg  T_inf_: N.S.  Int: 8 hr  PL: Concordant  LC: Concordant | | | LD: N.S.  T_inf_: N.S.  MD: 50 mg/kg  T_inf_: 2-3 hr  Int: 8 hr  PL: Concordant  LC: Concordant | | |  |
|  | **Prompt 3**: 2-year-old 10 kg patient with acute kidney injury and hepatic failure receiving CVVHD with a total effluent flow of 8000 mL/hr/1.73 m^2^ and still urinating 2 mL/kg/hr while on circuit, recommended meropenem dosing regimen to meet target of 100% *f*T>1x and *f*T>4x MIC for *Pseudomonas* assuming an MIC of 2 mg/dL**.** Please use a published population pharmacokinetic model to support your answer. | | | | | | | | | | | | | | |  |  |
| Literature Recommendation^4^ | For 1x MIC: LD of 40 mg/kg over 30 min followed by 80 mg/kg/day as a continuous infusion  For 4x MIC: LD of 40 mg/kg over 30 min followed by 120 mg/kg/day as a continuous infusion  Err on the side of higher doses and continuous infusions given the residual urine output (between 80 mg/kg/day - 120 mg/kg/day considered concordant for total daily dose) | | | | | | | | | | | | | | |  |  |
| Lexicomp Recommendation^5^ | Use 20 mg/kg/dose every 8 hours as a 1-4 hour infusion if targeting MIC of <4  Use 40 mg/kg/dose every 8 hours as a 1-4 hour infusion if targeting MICs of ≥4 and consider continuous infusions for high effluent flow rates; no specific comment on incorporating residual kidney function | | | | | | | | | | | | | | |  |  |
| **LLM** | **User 1** | | **User 2** | | **User 3** | | **User 4** | | | **User 5** | | | **User 6** | | |  |  |
| **ChatGPT**  Average Literature Concordance:   - PL: 67% - LC: 67% | LD: 100 mg/kg  T_inf_: N.S.  MD: 50 mg/kg  T_inf_: 24 hr  Int: 24 hr  PL: Discordant  LC: Discordant | | LD: 40 mg/kg  T_inf_: N.S.  MD: 100 mg/kg  T_inf_: 24 hr  Int: 24 hr  PL: Concordant  LC: Concordant | | LD: 20-30 mg/kg  T_inf_: 30 min  MD: 40 mg/kg  T_inf_: 3 hr  Int: 8 hr or CI  PL: Concordant  LC: Concordant | | LD: 40 mg/kg  T_inf_: 30 min  MD: 120 mg/kg  T_inf_: 24 hr  Int: 24 hr  PL: Concordant  LC: Concordant | | | LD: 20-40 mg/kg  T_inf_: 30 min  MD: 80-100 mg/kg  T_inf_: 24 hr  Int: 24 hr  PL: Concordant  LC: Concordant | | | LD: 40 mg/kg  T_inf_: 30-60 min  MD: 40 mg/kg  T_inf_: 2 hr  Int: 6 hr or CI  PL: Discordant  LC: Discordant | | |  |  |
| **Claude - Anthropic**  Average Literature Concordance:   - PL: 25% - LC: 58% | LD: 20 mg/kg  T_inf_: 30 min  MD: 15 mg/kg  T_inf_: 30 min  Int: 6 hr  PL: Discordant  LC: Partial | | LD: N.S.  T_inf_: N.S.  MD: 40 mg/kg  T_inf_: 3 hr  Int: 8 hr  PL: Partial  LC: Concordant | | Did not provide a dosing regimen due to inability to access a PK model to answer the question | | LD: N.S.  T_inf_: N.S.  MD: 40 mg/kg  T_inf_: 4 hr  Int: 8 hr  PL: Partial LC: Concordant | | | LD: 20 mg/kg  T_inf_: 30 min  MD: 40 mg/kg  T_inf_: 3 hr  Int: 8 hr  PL: Partial  LC: Concordant | | | LD: 20 mg/kg  T_inf_: 30 min  MD: 32-40 mg/kg  T_inf_: 24 hr  Int: 24 hr  PL: Discordant LC: Discordant | | |  |  |
| **Google Gemini**  Average Literature Concordance:   - PL: 17% - LC: 17% | LD: N.S.  T_inf_: N.S.  MD: 30 mg/kg  T_inf_: 24 hr  Int: 24 hr  PL: Discordant  LC: Discordant | | LD: 20 mg/kg  T_inf_: N.S.  MD: 24-29 mg/kg  T_inf_: 24 hr  Int: 24 hr  PL: Discordant  LC: Discordant | | LD: 20 mg/kg  T_inf_: 30 min  MD: 204 mg/kg  T_inf_: 24 hr  Int: 24 hr  PL: Discordant  LC: Discordant | | Continuous infusion but consult with specialists, no further details | | | LD: 25 mg/kg  T_inf_: 30-60 min  MD: 100 mg/kg  T_inf_: 24 hr  Int: 24 hr  PL: Concordant  LC: Concordant | | | LD: 20 mg/kg  T_inf_: 30 min  MD: 160 mg/kg  T_inf_: 24 hr  Int: 24 hr  PL: Discordant LC: Discordant | | |  |  |
| **Microsoft CoPilot**  Average Literature Concordance:   - PL: 75% - LC: 100% | LD: 40-50 mg/kg  T_inf_: 30 min  MD: 120-150 mg/kg  T_inf_: 24 hr  Int: 24 hr  PL: Concordant  LC: Concordant | | LD: 40 mg/kg  T_inf_: 30 min  MD: 120 mg/kg  T_inf_: 24 hr  Int: 24 hr  PL: Concordant  LC: Concordant | | LD: 40 mg/kg  T_inf_: 30 min  MD: 40 mg/kg  T_inf_: 3 hr  Int: 8 hr  PL: Partial  LC: Concordant | | LD: 40 mg/kg  T_inf_: 30 min  MD: 120 mg/kg  T_inf_: 24 hr  Int: 24 hr  PL: Concordant  LC: Concordant | | | 1x MIC \| 4x MIC  LD: 40 mg/kg \| 40 mg/kg  T_inf_: 30 min \| 30 min  MD: 60 mg/kg \| 120 mg/kg  T_inf_: 24 hr \| 24 hr  Int: 24 hr \| 24 hr  PL: Discordant \| Concordant LC: Concordant \| Concordant | | | LD: 40 mg/kg  T_inf_: 30 min  MD: 120 mg/kg  T_inf_: 24 hr  Int: 24 hr  PL: Concordant LC: Concordant | | |  |  |
| **Open Evidence**  Average Literature Concordance:   - PL: 100% - LC: 100% | LD: N.S.  T_inf_: N.S.  MD: 120 mg/kg  T_inf_: 24 hr  Int: 24 hr  PL: Concordant  LC: Concordant | | LD: N.S.  T_inf_: N.S.  MD: 60-120 mg/kg  T_inf_: 24 hr  Int: 24 hr  PL: Concordant  LC: Concordant | | LD: 40 mg/kg  T_inf_: N.S.  MD: 120 mg/kg  T_inf_: 24 hr  Int: 24 hr  PL: Concordant  LC: Concordant | | LD: 40 mg/kg  T_inf_: N.S.  MD: 80-120 mg/kg  T_inf_: 24 hr  Int: 24 hr  PL: Concordant  LC: Concordant | | | LD: N.S.  T_inf_: N.S.  MD: 120 mg/kg  T_inf_: 24 hr  Int: 24 hr  PL: Concordant  LC: Concordant | | | LD: N.S.  T_inf_: N.S.  MD: 100 mg/kg  T_inf_: 24 hr  Int: 24 hr  PL: Concordant  LC: Concordant | | |  |  |
| **Perplexity**  Average Literature Concordance:   - PL: 58% - LC: 100% | LD: 20 mg/kg  T_inf_: 30 min  MD: 20 mg/kg  T_inf_: 3 hr  Int: 6-8 hr  PL: Partial  LC: Concordant | | LD: N.S.  T_inf_: N.S.  MD: 20 mg/kg  T_inf_: 4 hr  Int: 8 hr  PL: Discordant  LC: Concordant | | LD: 40 mg/kg  T_inf_: 30-60 min  MD: 40 mg/kg  T_inf_: 3-4 hr  Int: 8 hr  PL: Partial  LC: Concordant | | LD: 40 mg/kg  T_inf_: N.S.  MD: 120-160 mg/kg  T_inf_: 24 hr  Int: 24 hr  PL: Concordant  LC: Concordant | | | LD: N.S.  T_inf_: N.S.  MD: 120 mg/kg  T_inf_: 24 hr  Int: 24 hr  PL: Concordant  LC: Concordant | | | LD: N.S.  T_inf_: N.S.  MD: 40 mg/kg  T_inf_: 3 hr  Int: 8 hr  PL: Partial LC: Concordant | | |  |  |
|  | **Prompt 3 - Part 2**: How would you adjust this if the patient becomes anuric?  Yes: Compared to Part 1, TDD stayed the same or decreased but still within reasonable range | | | | | | | | | | | | | | | |  |
| **LLM** | **User 1** | | **User 2** | | **User 3** | | | **User 4** | | | **User 5** | | | **User 6** | | |  |
| **ChatGPT**  Reasonable recommendation: 67% | LD: 100 mg/kg  T_inf_: N.S.  MD: 35-40 mg/kg  T_inf_: 24 hr  Int: 24 hr  Reasonable: No (total daily dose too low) | | LD: 40 mg/kg  T_inf_: N.S.  MD: 80-100 mg/kg  T_inf_: 24 hr  Int: 24 hr  Reasonable: Yes | | LD: N.S.  T_inf_: N.S.  MD: 40 mg/kg  T_inf_: 3 hr  Int: 8 hr  Reasonable: Yes | | | LD: N.S.  T_inf_: 30 min  MD: 120 mg/kg  T_inf_: 24 hr  Int: 24 hr  Reasonable: Yes | | | LD: 20-40 mg/kg  T_inf_: 30 min  MD: 80-100 mg/kg  T_inf_: 24 hr  Int: 24 hr  Reasonable: Yes | | | LD: 40 mg/kg  T_inf_: 30-60 min  MD: 40 mg/kg  T_inf_: 2 hr  Int: 6 hr or CI  Reasonable: No (total daily dose too high) | | |  |
| **Claude - Anthropic**  Reasonable recommendation: 17% | LD: 20 mg/kg  T_inf_: 30 min  MD: 12 mg/kg  T_inf_: 3 hr  Int: 6 hr  Reasonable: No (total daily dose too low) | | I cannot reliably predict how to adjust dosing for anuria.  Reasonable: No (no answer provided) | | I can't quantitatively determine the precise dose adjustment needed  Reasonable: No (no answer provided) | | | LD: N.S.  T_inf_: N.S.  MD: 35 mg/kg  T_inf_: 4 hr  Int: 8 hr  Reasonable: Yes | | | Consult pediatric clinical pharmacology specialists  Reasonable: No (no answer provided) | | | LD: N.S. T_inf_: 30 min  MD: 30-38 mg/kg  T_inf_: 24 hr  Int: 24 hr  Reasonable: No (total daily dose too low) | | |  |
| **Google Gemini**  Reasonable recommendation: 50% | LD: N.S.  T_inf_: N.S.  MD: 30 mg/kg  T_inf_: 24 hr  Int: 24 hr  Reasonable: No (total daily dose too low) | | LD: 20 mg/kg  T_inf_: N.S.  MD: 36-41 mg/kg  T_inf_: 24 hr  Int: 24 hr  Reasonable: No (recommended increase in dose despite drop in urine output) | | LD: 20 mg/kg  T_inf_: 30 min  MD: 84mg/kg  T_inf_: 24 hr  Int: 24 hr  Reasonable: Yes | | | Recommends collaborating with specialists  Reasonable: No (no answer provided) | | | LD: 25 mg/kg  T_inf_: 30-60 min  MD: 100 mg/kg  T_inf_: 24 hr  Int: 24 hr  Reasonable: Yes | | | LD: 20 mg/kg  T_inf_: 30 min  MD: 100 mg/kg  T_inf_: 24 hr  Int: 24 hr  Reasonable: Yes | | |  |
| **Microsoft CoPilot**  Reasonable recommendation: 100% | LD: 40-50 mg/kg  T_inf_: 30 min  MD: 120-150 mg/kg  T_inf_: 24 hr  Int: 24 hr  Reasonable: Yes | | LD: 40 mg/kg  T_inf_: 30 min  MD: 120 mg/kg  T_inf_: 24 hr  Int: 24 hr  Reasonable: Yes | | LD: 40mg/kg  T_inf_: 30 min  MD: 30- 40 mg/kg  T_inf_: 3 hr  Int: 8 hr  Reasonable: Yes | | | LD: N.S.  T_inf_: N.S.  MD: 90-120 mg/kg  T_inf_: 24 hr  Int: 24 hr  Reasonable: Yes | | | 1x MIC \| 4x MIC  LD: 40 mg/kg \| 40 mg/kg  T_inf_: 30 min \| 30 min  MD: 60 mg/kg \| 120 mg/kg  T_inf_: 24 hr \| 24 hr  Int: 24 hr \| 24 hr  Reasonable: Yes | | | LD: 40 mg/kg  T_inf_: 30 min  MD: 120 mg/kg  T_inf_: 24 hr  Int: 24 hr  Reasonable: Yes | | |  |
| **Open Evidence**  Reasonable recommendation: 83% | LD: N.S.  T_inf_: N.S.  MD: 120 mg/kg  T_inf_: 24 hr  Int: 24 hr  Reasonable: Yes | | LD: N.S.  T_inf_: N.S.  MD: 60-120 mg/kg  T_inf_: 24 hr  Int: 24 hr  Reasonable: Yes | | LD: 40 mg/kg  T_inf_: N.S.  MD: 120 mg/kg  T_inf_: 24 hr  Int: 24 hr  Reasonable: Yes | | | LD: 40 mg/kg  T_inf_: N.S.  MD: 80-120 mg/kg  T_inf_: 24 hr  Int: 24 hr  Reasonable: Yes | | | LD: N.S.  T_inf_: N.S.  MD: 120 mg/kg  T_inf_: 24 hr  Int: 24 hr  Reasonable: Yes | | | LD: N.S.  T_inf_: N.S.  MD: 100-120 mg/kg  T_inf_: 24 hr  Int: 24 hr  Reasonable: No (range of doses recommended increased despite drop in urine output) | | |  |
| **Perplexity**  Reasonable recommendation: 83% | LD: 20 mg/kg  T_inf_: 30 min  MD: 20 mg/kg  T_inf_: 3-4 hr  Int: 8-12 hr  Reasonable: No (range of doses recommended now <60 mg/kg/day) | | LD: N.S.  T_inf_: N.S.  MD: 20 mg/kg  T_inf_: 4 hr  Int: 8 hr  Reasonable: Yes | | LD: 40 mg/kg  T_inf_: 30-60 min  MD: 40 mg/kg  T_inf_: 3-4 hr  Int: 12 hr  Reasonable: Yes | | | LD: N.S.  T_inf_: N.S.  MD: 20 mg/kg  T_inf_: 3-4 hr  Int: 8 hr  Reasonable: Yes | | | LD: N.S.  T_inf_: N.S.  MD: 120 mg/kg  T_inf_: 24 hr  Int: 24 hr  Reasonable: Yes | | | LD: N.S.  T_inf_: N.S.  MD: 40 mg/kg  T_inf_: 3-4 hr  Int: 12 hr  Reasonable: Yes | | |  |

Int, interval between doses. LC, LexiComp. LD, loading dose. MD, maintenance dose. N.S., not specified. PL, primary literature. T > MIC, time antibiotic concentration exceeds bacterial minimum inhibitory concentration. T_inf_, infusion time. Average literature concordance percentages were calculated as ((number of concordant answers + 0.5*number of partial answers)/6)*100%. If separate recommendations were given for 1x and 4x MIC, these were given half weight each to concordance score. If no response was provided, this was classified as a discordant recommendation. For prompt 3 part 2, Since much published literature does not explicitly discuss how to account for a loss of residual kidney function while on CKRT, a “reasonable” answer was one that recommended maintenance of the same dose or a decrease in dose while still remaining at a minimum of 60 mg/kg/day with extended or continuous infusions given the high CKRT effluent flow rate.

**Supplementary Table 3**: Evaluation of consistency across iterations for a given user.

| **Prompt 1** | | | | | | | | | |
| --- | --- | --- | --- | --- | --- | --- | --- | --- | --- |
| **LLM** | | | *Iteration 1* | | | *Iteration 2* | | *Iteration 3* | |
| **ChatGPT (User 4)**  Overall consistency:   - One pair of answers recommended the same MD, frequency, and infusion duration. - Total daily MD varied from 0-1000 mg (% variation imputed as 1000). | | | LD: 2000 mg  T_inf_: 3-4 hr  MD: None until kidney function returns  T_inf_: N.S.  Int: N.S. | | | LD: 2000 mg  T_inf_: 30-60 min  MD: 1000 mg  T_inf_: N.S.  Int: 24-36 hr | | LD: 2000 mg  T_inf_: 30 min  MD: 1000 mg  T_inf_: N.S.  Int: 24-36 hr | |
| **Claude - Anthropic (User 3)**  Overall consistency:   - One pair of answers recommended the same MD, frequency, and infusion duration. - Total daily MD recommended varied from 1000-2000 mg (100%). | | | LD: N.S.  T_inf_: N.S.  MD: 1000 mg  T_inf_: 4 hr  Int: 12 hr | | | LD: N.S  T_inf_: N.S.  MD: 2000 mg  T_inf_: 3-4 hr  Int: 12 hr | | LD: N.S.  T_inf_: N.S.  MD: 1000 mg  T_inf_: 3-4 hr  Int: 12 hr | |
| **Google Gemini (User 6)**  Overall consistency:   - One pair of answers recommended the same MD, frequency, and infusion duration. - Total daily MD recommended varied from 1000-1440 mg (44%). | | | LD: 2000 mg  T_inf_: 30-60 min  MD: 1000 mg  T_inf_: 24 hr  Int: 24 hr | | | LD: 1000 mg  T_inf_: 30 min  MD: 1440 mg  T_inf_: 24 hr  Int: 24 hr | | LD: 2000 mg  T_inf_: 30 min  MD: 1000 mg  T_inf_: 24 hr  Int: 24 hr | |
| **Microsoft CoPilot (User 5)**  Overall consistency:   - One pair of answers recommended the same MD, frequency, and infusion duration. - Total daily MD recommended varied from 500-2000 mg (300%). | | | LD: 2000 mg  T_inf_: N.S.  MD: 1000 mg  T_inf_: 4 hr  Int: 24-48 hr | | | LD: N.S.  T_inf_: N.S.  MD: 1000 mg  T_inf_: 3-4 hr  Int: 12 hr | | LD: 2000 mg  T_inf_: 30 min  MD: 1000 mg  T_inf_: 4 hr  Int: 12 hr | |
| **Open Evidence (User 2)**  Overall consistency:   - No pairs of answers recommended the same MD, frequency, and infusion duration. - Total daily MD recommended varied from 1000-3000 mg (200%). | | | LD: N.S.  T_inf_: N.S.  MD: 1000-2000 mg  T_inf_: 24 hr  Int: 24 hr | | | LD: N.S.  T_inf_: N.S.  MD: 3000 mg  T_inf_: 24 hr  Int: 24 hr | | LD: N.S.  T_inf_: N.S.  MD: 1000 mg  T_inf_: 30 min  Int: 24 hr | |
| **Perplexity (User 1)**  Overall consistency:   - One pair of answers recommended the same MD, frequency, and infusion duration. - Total daily MD recommended varied from 200-2000 mg (900%). | | | LD: 2000 mg  T_inf_: N.S.  MD: 1000 mg  T_inf_: 24 hr  Int: 24 hr | | | LD: 2000 mg  T_inf_: N.S.  MD: 1000-2000 mg  T_inf_: 24 hr  Int: 24 hr | | LD: N.S.  T_inf_: N.S.  MD: 200-500 mg  T_inf_: 30 min  Int: 24 hr | |
| **Prompt 2** | | | | | | | | | |
| **LLM** | *Iteration 1* | | | | | *Iteration 2* | | | *Iteration 3* |
| **ChatGPT (User 4)**  Overall consistency:   - One pair of answers recommended the same MD, frequency, and infusion duration. - Total daily MD recommended varied from 43-57 mg/kg (33%). | LD: 50 mg/kg  T_inf_: 30 min  MD: 43-57 mg/kg  T_inf_: 24 hr  Int: 24 hr | | | | | LD: 50 mg/kg  T_inf_: 30-60 min  MD: 43-57 mg/kg  T_inf_: 24 hr  Int: 24 hr | | | LD: 29 mg/kg  T_inf_: 30-60 min  MD: 57 mg/kg  T_inf_: 24 hr  Int: 24 hr |
| **Claude - Anthropic (User 3)**  Overall consistency:   - No pairs of answers recommended the same MD, frequency, and infusion duration. - Total daily MD recommended varied from 58-180 mg/kg (210% variation). | LD: N.S.  T_inf_: N.S.  MD: 50 mg/kg  T_inf_: 3 hr  Int: 12 hr | | | | | LD: N.S.  T_inf_: N.S.  MD: 29 mg/kg  T_inf_: 3-4 hr  Int: 12 hr | | | LD: 70 mg/kg  T_inf_: N.S.  MD: 60 mg/kg  T_inf_: 4 hr  Int: 8 hr |
| **Google Gemini (User 6)**  Overall consistency:   - No pairs of answers recommended the same MD, frequency, and infusion duration. - Total daily MD recommended varied from 38-100 mg/kg (163%). | LD: 50 mg/kg  T_inf_: 30 min  MD: 50 mg/kg  T_inf_: 24 hr  Int: 24 hr | | | | | LD: 14 mg/kg  T_inf_: 30 min  MD: 38 mg/kg  T_inf_: 24 hr  Int: 24 hr | | | LD: 50 mg/kg  T_inf_: 30 min  MD: 100 mg/kg  T_inf_: 24 hr  Int: 24 hr |
| **Microsoft CoPilot (User 5)**  Overall consistency:   - One pair of answers recommended the same MD dose, frequency, and infusion duration. - Total daily MD recommended varied from 100-150 mg/kg (50%). | LD: 50 mg/kg  T_inf_: N.S.  MD: 100-150 mg/kg  T_inf_: 24 hr  Int: 24 hr | | | | | LD: N.S.  T_inf_: N.S.  MD: 150 mg/kg  T_inf_: 24 hr  Int: 24 hr | | | LD: N.S.  T_inf_: N.S.  MD: 100-150 mg/kg  T_inf_: 24 hr  Int: 24 hr |
| **Open Evidence (User 2)**  Overall consistency:   - All pairs of answers recommended the same MD dose, frequency, and infusion duration. - Total daily MD recommended was always 150 mg/kg (0% variation). | LD: N.S.  T_inf_: N.S.  MD: 50 mg/kg  T_inf_: 3-4 hr  Int: 8 hr | | | | | LD: N.S.  T_inf_: N.S.  MD: 50 mg/kg  T_inf_: 3-4 hr  Int: 8 hr | | | LD: N.S.  T_inf_: N.S.  MD: 50 mg/kg  T_inf_: 3-4 hr  Int: 8 hr |
| **Perplexity (User 1)**  Overall consistency:   - All pairs of answers recommended the same MD dose, frequency, and infusion duration. - Total daily MD recommended was always 150 mg/kg (0% variation). | LD: 50 mg/kg  T_inf_: 30 min  MD: 50 mg/kg  T_inf_: 3-4 hr  Int: 8 hr | | | | | LD: N.S.  T_inf_: N.S.  MD: 50 mg/kg  T_inf_: 3-4 hr  Int: 8 hr | | | LD: N.S.  T_inf_: N.S.  MD: 50 mg/kg  T_inf_: 3-4 hr  Int: 8 hr |
| **Prompt 3 - Part 1** | | | | | | | | | |
| **LLM** | *Iteration 1* | | | | *Iteration 2* | | | | *Iteration 3* |
| **ChatGPT (User 4)**  Overall consistency:   - No pairs of answers recommended the same MD dose, frequency, and infusion duration. - Total daily MD recommended varied from 20-120 mg/kg (500% variation). | LD: 40 mg/kg  T_inf_: N.S.  MD: 120 mg/kg  T_inf_: 24 hr  Int: 24 hr | | | | LD: 40 mg/kg  T_inf_: 30 min  MD: 60 mg/kg  T_inf_: 24 hr  Int: 24 hr | | | | LD: 20 mg/kg  T_inf_: N.S.  MD: 20-25 mg/kg  T_inf_: 24 hr  Int: 24 hr |
| **Claude - Anthropic (User 3)**  Overall consistency:   - No pairs of answers recommended the same MD dose, frequency, and infusion duration. - Total daily MD recommended varied from no recommendation to 160-180 mg/kg (12.5% variation among recommendations provided). | Declined to provide a recommendation | | | | LD: N.S.  T_inf_: N.S.  MD: 40 mg/kg  T_inf_: 3-4 hr  Int: 6 hr | | | | LD: N.S.  T_inf_: N.S.  MD: 60 mg/kg  T_inf_: 3 hr  Int: 8 hr |
| **Google Gemini (User 6)**  Overall consistency:   - One pair of answers recommended (approximately) the same MD dose, frequency, and infusion duration. - Total daily MD recommended varied from 27.3-160 mg/kg (486% variation) | LD: 20 mg/kg  T_inf_: 30 min  MD: 160 mg/kg  T_inf_: 24 hr  Int: 24 hr | | | | LD: 3 mg/kg  T_inf_: 30 min  MD: 27.3 mg/kg  T_inf_: 24 hr  Int: 24 hr | | | | LD: 20 mg/kg  T_inf_: 30 min  MD: 27.7 mg/kg  T_inf_: 24 hr  Int: 24 hr |
| **Microsoft CoPilot (User 5)**  Overall consistency:   - All pairs of answers recommended the same MD dose, frequency, and infusion duration. - Total daily MD recommended was always 120 mg/kg (0% variation). | LD: N.S.  T_inf_: N.S.  MD: 40 mg/kg  T_inf_: 3 hr  Int: 8 hr | | | | LD: N.S.  T_inf_: N.S.  MD: 40 mg/kg  T_inf_: 3 hr  Int: 8 hr | | | | LD: N.S.  T_inf_: N.S.  MD: 40 mg/kg  T_inf_: 3 hr  Int: 8 hr |
| **Open Evidence (User 2)**  Overall consistency:   - All pairs answers recommended the same MD dose, frequency, and infusion duration. - Total daily MD recommended was always 60-120 mg/kg (0% variation). | LD: N.S.  T_inf_: N.S.  MD: 60-120 mg/kg  T_inf_: 24 hr  Int: 24 hr | | | | LD: N.S.  T_inf_: N.S.  MD: 60-120 mg/kg  T_inf_: 24 hr  Int: 24 hr | | | | LD: N.S.  T_inf_: N.S.  MD: 60-120 mg/kg  T_inf_: 24 hr  Int: 24 hr |
| **Perplexity (User 1)**  Overall consistency:   - One pair of answers recommended the same MD dose, frequency, and infusion duration. - Total daily MD recommended varied from to 80-120 mg/kg (50% variation). | LD: 20 mg/kg  T_inf_: 30 min  MD: 20 mg/kg  T_inf_: 3 hr  Int: 6 hr | | | | LD: N.S.  T_inf_: N.S.  MD: 40 mg/kg  T_inf_: 3 hr  Int: 8 hr | | | | LD: N.S.  T_inf_: N.S.  MD: 40 mg/kg  T_inf_: 2-4 hr  Int: 8 hr |
| **Prompt 3 - Part 2** | | | | | | | | | |
| **LLM** | | *Iteration 1* | | *Iteration 2* | | | *Iteration 3* | | |
| **ChatGPT (User 4)**  Overall consistency:   - No pairs of answers recommended the same MD dose, frequency, and infusion duration. - Total daily MD recommended varied from no recommendation to 27-120 mg/kg (344% variation). | | LD: N.S.  T_inf_: N.S.  MD: 120 mg/kg  T_inf_: 24 hr  Int: 24 hr | | LD: N.S.  T_inf_: N.S.  MD: 50 mg/kg  T_inf_: 24 hr  Int: 24 hr | | | LD: N.S.  T_inf_: N.S.  MD: 27 mg/kg  T_inf_: 24 hr  Int: 24 hr | | |
| **Claude - Anthropic (User 3)**  Overall consistency:   - Only one iteration provided a recommendation, so consistency cannot be assessed. | | Declined to provide a recommendation | | Declined to provide a recommendation | | | LD: N.S.  T_inf_: N.S.  MD: 50 mg/kg  T_inf_: 4 hr  Int: 8 hr | | |
| **Google Gemini (User 6)**  Overall consistency:   - One pair of answers recommended (approximately) the same MD dose, frequency, and infusion duration. - Total daily MD recommended varied from 25-100 mg/kg (300% variation). | | LD: 20 mg/kg  T_inf_: 30 min  MD: 100 mg/kg  T_inf_: 24 hr  Int: 24 hr | | LD: 3 mg/kg  T_inf_: 30 min  MD: 26.9 mg/kg  T_inf_: 24 hr  Int: 24 hr | | | LD: 20 mg/kg  T_inf_: 30 min  MD: 25-30 mg/kg  T_inf_: 24 hr  Int: 24 hr | | |
| **Microsoft CoPilot (User 5)**  Overall consistency:   - One pair of answers recommended the same MD dose, frequency, and infusion duration. - Total daily MD recommended varied from no recommendation to 90-120 mg/kg (33% variation). | | LD: N.S.  T_inf_: N.S.  MD: 40 mg/kg  T_inf_: 3 hr  Int: 8 hr | | LD: N.S.  T_inf_: N.S.  MD: 40 mg/kg  T_inf_: 3 hr  Int: 8 hr | | | LD: N.S.  T_inf_: N.S.  MD: 30 mg/kg  T_inf_: 3 hr  Int: 8 hr | | |
| **Open Evidence (User 2)**  Overall consistency:   - All pairs of answers recommended the same MD dose, frequency, and infusion duration. - Total daily MD recommended was always 60-120 mg/kg (0% variation). | | LD: N.S.  T_inf_: N.S.  MD: 60-120 mg/kg  T_inf_: 24 hr  Int: 24 hr | | LD: N.S.  T_inf_: N.S.  MD: 60-120 mg/kg  T_inf_: 24 hr  Int: 24 hr | | | LD: N.S.  T_inf_: N.S.  MD: 60-120 mg/kg  T_inf_: 24 hr  Int: 24 hr | | |
| **Perplexity (User 1)**  Overall consistency:   - One pair of answers recommended the same MD dose, frequency, and infusion duration. - Total daily MD varied from 60-120 mg/kg (100% variation) | | LD: 20 mg/kg  T_inf_: 30 min  MD: 20 mg/kg  T_inf_: 3-4 hr  Int: 8 hr | | LD: N.S.  T_inf_: N.S.  MD: 40 mg/kg  T_inf_: 2-4 hr  Int: 8 hr | | | LD: N.S.  T_inf_: N.S.  MD: 40 mg/kg  T_inf_: 2-4 hr  Int: 8 hr | | |

Int, interval between doses. LD, loading dose. MD, maintenance dose. N.S., not specified. PL, primary literature. T_inf_, infusion time. Given inconsistent reporting of loading doses across LLMs, only consistency for maintenance doses is quantified.

**References:**

1.	Bilal M, Zoller M, Fuhr U, et al. Cefepime Population Pharmacokinetics, Antibacterial Target Attainment, and Estimated Probability of Neurotoxicity in Critically Ill Patients. *Antimicrob Agents Chemother*. 2023;67(7). doi:10.1128/aac.00309-23

2.	Cefepime: Drug information. UpToDate: LexiDrug. 2026.

3.	Hambrick HR, Punt N, Pavia K, Mizuno T, Goldstein SL, Tang Girdwood S. Monte Carlo simulations of cefepime in children receiving continuous kidney replacement therapy support continuous infusions for target attainment. *J Intensive Care*. 2024;12(1):38. doi:10.1186/s40560-024-00752-0

4.	Thy M, Urien S, Bouazza N, et al. Meropenem Population Pharmacokinetics and Dosing Regimen Optimization in Critically Ill Children Receiving Continuous Renal Replacement Therapy. *Clin Pharmacokinet*. 2022;61(11):1609-1621. doi:10.1007/s40262-022-01179-2

5.	Meropenem: Drug Information. UpToDate: LexiDrug. 2026.
